# Characterization of Polylactic Acid Biocomposites Filled with Native Starch Granules from *Dioscorea remotiflora* Tubers

**DOI:** 10.3390/polym16070899

**Published:** 2024-03-25

**Authors:** Yokiushirdhilgilmara Estrada-Girón, Víctor Vladimir Amílcar Fernández-Escamilla, Angelina Martín-del-Campo, Rubén González-Nuñez, Gonzalo Canché-Escamilla, Jorge Uribe-Calderón, Nancy Tepale, Jacobo Aguilar, Francisco Javier Moscoso-Sánchez

**Affiliations:** 1 Departamento de Ingeniería Química, Centro Universitario de Ciencias Exactas e Ingenierías, Universidad de Guadalajara, Blvd. Marcelino García Barragán 1421, Col. Olímpica, Guadalajara 44430, Jalisco, Mexico; 2 Departamento de Ciencias Tecnológicas, Centro Universitario de la Ciénega, Universidad de Guadalajara, Av. Universidad 1115, Col. Lindavista, Ocotlán 47820, Jalisco, Mexico; 3 Unidad Académica de Materiales, Centro de Investigación Científica de Yucatán, Calle 43 No. 130, Chuburná de Hidalgo, Mérida 97205, Yucatán, Mexico; 4 Facultad de Ingeniería Química, Benemérita Universidad Autónoma de Puebla, Av. San Claudio y 18 Sur S/N, Col. San Manuel, Puebla 72570, Puebla, Mexico; 5 Departamento de Química, Centro Universitario de Ciencias Exactas e Ingenierías, Universidad de Guadalajara, Blvd. Marcelino García Barragán 1421, Col. Olímpica, Guadalajara 44430, Jalisco, Mexico

**Keywords:** mountain’s yam starch, PLA blending, biocomposite characterization, biodegradable materials, low-cost bioplastics

## Abstract

Biocomposites were fabricated utilizing polylactic acid (PLA) combined with native starch sourced from mountain’s yam (*Dioscorea remotiflora* Knuth), an underexplored tuber variety. Different starch compositions (7.5, 15.0, 22.5, and 30.0 wt.%) were blended with PLA in a batch mixer at 160 °C to produce PLA/starch biocomposites. The biocomposites were characterized by analyzing their morphology, particle size distribution, thermal, X-ray diffraction (XDR), mechanical, and dynamic mechanical (DMA) properties, water absorption behavior, and color. The results showed that the amylose content of *Dioscorea remotiflora* starch was 48.43 ± 1.4%, which corresponds to a high-amylose starch (>30% of amylose). Particle size analysis showed large z-average particle diameters (*D_z0_*) of the starch granules (30.59 ± 3.44 μm). Scanning electron microscopy (SEM) images showed oval-shaped granules evenly distributed throughout the structure of the biocomposite, without observable agglomeration or damage to its structure. XDR and DMA analyses revealed an increase in the crystallinity of the biocomposites as the proportion of the starch increased. The tensile modulus (E) underwent a reduction, whereas the flexural modulus (E_flex_) increased with the amount of starch incorporated. The biocomposites with the highest E_flex_ were those with a starch content of 22.5 wt.%, which increased by 8.7% compared to the neat PLA. The water absorption of the biocomposites demonstrated a higher uptake capacity as the starch content increased. The rate of water absorption in the biocomposites followed the principles of Fick’s Law. The novelty of this work lies in its offering an alternative for the use of high-amylose mountain’s yam starch to produce low-cost bioplastics for different applications.

## 1. Introduction

The advancement of biodegradable materials as substitutes for conventional plastics that generate a greater impact on the environment has become crucial. From this perspective, the latest research has assessed the replacement of petroleum derivatives with biodegradable components to create more eco-friendly materials. In this regard, various types of materials, such as polyethylene (PE), low-density polyethylene (LDPE), polycaprolactone (PCL), and polylactic acid (PLA), have been evaluated in combination with organic residues or plant bioproducts to increase the rate of biodegradability of purely polymeric materials [1,2,3].

In particular, PLA, despite its many advantages (biodegradability transparency, good mechanical properties, and safety for food packaging), has limited uses due to its high cost [4,5,6]. Therefore, PLA is often blended with other low-cost biopolymers or biofillers that modify the properties of the resulting composites as a function of the nature and composition of the added materials [6,7]. Further, extensive research has been reported elsewhere on the use of PLA reinforced with natural fibers (agave, henequen, or sisal, among others) to obtain composite materials of improved biodegradability, density, strength, and moduli [8,9,10]. Moreover, for application in food packaging, biocomposites with favorable mechanical properties and large crystallinity are required [11,12]. On the other hand, starch is another type of biopolymer of low cost and broad availability that is being used in the fabrication of biodegradable plastics [13,14]. Starch is a biodegradable polymer that can be processed in large quantities at a relatively low cost, it is easy to handle, and it can form film products of low oxygen permeability, with the main challenge of native starch being its fragility and hydrophilicity [15]. In this context, thermoplastic blends of PE, LDPE, PCL, and PLA with starch exhibit an increased biodegradation rate and a decrease in brittleness and rigidity when plasticized under heating and shearing; this results in a continuous phase forming a polymer melt that can be processed using traditional plastic processing techniques, such as extrusion and injection molding [16,17,18].

Among the most common sources of starch utilized to develop bioplastics are corn, potato, rice, wheat, or cassava; in addition, starch is also employed as an additive for different food, pharmaceutical, and industrial products [15,19]. Worldwide, these crops belong to the group of basic foods that form part of the daily diet, and their extensive exploitation could result in shortages. However, less exploited cultivars, such as mountain’s yam (*Dioscorea remotiflora* Kunth), are also excellent sources of starch and, at the same time, could increase the cultivar’s added value without compromising its availability. In this respect, the mountain’s yam is a wild native plant species that grows in the western regions of Mexico and that is mainly consumed by the local population as a cooked vegetable. This type of tuber contains approximately 85% carbohydrates, which is nearly like that of potatoes (*Solanum tuberosum*) [20]; therefore, it may constitute an important source of starch (22.1%) [21], currently utilized for scarce industrial purposes, making it an ideal source of starch without compromising the supply of staple crops. The proximate composition analysis of mountain’s yam (*Dioscorea remotiflora* Kunth) has been reported elsewhere [21]. More importantly, as is shown here, the amylose content of *Dioscorea remotiflora* starch is higher than that of conventional starch sources, i.e., potato, which contains 20% [22]. Amylose is a linear polymer with α(1→4)-D-glycosidic bonds [23], which determines the functional and physicochemical properties of starch [24]. Starches with a high amylose content are more resistant and, in foods, are less digestible than regular starches [25], and they produce firm gels with film-forming properties, which are ideal for use as bioplastics [26] and improve the mechanical properties of PLA biocomposites [27,28]. Moreover, as pointed out elsewhere [29], amylose acts as a plasticizer, modifying the properties of starch.

On the other hand, PLA and starch are incompatible, as they are hydrophobic and hydrophilic materials, respectively. In this regard, efforts to increase the compatibility between starch and PLA have been forwarded. To enhance the compatibility between starch and PLA, researchers have focused on using thermoplastic starch in the presence of plasticizers like water, glycerol, and sorbitol, and coupling agents such as polyethylene glycol, maleic anhydride, acrylic acid, polycaprolactone, and epoxidized soybean oil [30]. The latter has led to the development of more efficient and versatile biodegradable materials [31,32,33,34,35]. In addition, grafting techniques have been used, including glycidyl methacrylate-grafted poly (ethylene octane) [36,37], glycidyl methacrylate-grafted PLA [38], PLA-grafted starch [39], butyl-etherification [27], or methylene diphenyl diisocyanate (MDI) [40,41]. However, the latter is a toxic and non-biodegradable agent [42], moving away from the concept of green chemistry, which seeks to produce environmentally friendly biomaterials, for example, for drug-delivery applications [43]. Thus, the modification of the starch without the use of hazardous substances is preferred to increase the compatibility between the starch and PLA, such as the cross-linking of starch with sodium tripolyphosphate (STPP), a non-toxic polyanion [44], and citric acid [45], which creates intermolecular bonds to increase its hydrophobicity due to the incorporation of ester groups. Furthermore, the functionalization of starch for food applications permitted by the FDA (Food and Drug Administration, USA) has been carried out through the acetylation with acetic anhydride or acetic acid, allowing only low percentages of acetyl groups in starch (2.5 g/100 g) [46]. However, the latter reaction involves the use of sodium hydroxide as an activator. Thus, these physicochemical modifications of starch destroy and break down the structure of the starch granules, causing irreversible changes in the properties of starch, such as biodegradability, biocompatibility, and toxicity, among others [47], rendering them unviable for medical applications [48]. Furthermore, in the presence of water, this causes the linear amylose chains to leach out of the granules, modifying their physicochemical properties [46]. Consequently, the alternative of using native starch granules, without chemical modification, in the preparation of PLA composites has been less explored, and only a few recent works have analyzed the interactions between PLA and starch granules and their effect on the mechanical and thermal properties [47,48,49].

In this work, we examined the flexural and tensile strength, storage, loss modulus, (tan δ) = G″/G′, water absorption kinetic behavior, morphology, color, crystallinity, and thermal properties of PLA/starch biocomposites containing an underutilized source of native (non-chemically modified) high-amylose starch from mountain’s yam (*Dioscorea remotiflora* Kunth). These biocomposites have a larger water uptake capacity and higher crystallinity than neat PLA. More importantly, the flexural modulus of these biocomposites increases with an increasing starch content until reaching the turnover mass ratio of starch granules to the PLA matrix of 22.5:77.5, after which the flexural modulus decreases at larger starch granule/PLA matrix weight ratios. Since the starch granules are larger (30.59 ± 3.44 μm) and have a higher amylose content (48.43 ± 1.4 %) than those extracted from conventional starch sources, these biocomposites have larger tensile strength values than PLA biocomposites prepared with starch granules of similar sizes but from different botanical sources with a low amylose content. To the best of our knowledge, and despite the large number of studies on PLA biocomposites, there is a lack of research on mountain’s yam (*Dioscorea remotiflora* Kunth) as a source of reinforcing starch granules in PLA blends. This makes these biocomposites an alternative for the development of environmentally friendly materials with more sustainable methods.

## 2. Materials and Methods

### 2.1. Materials

PLA was acquired from Natureworks Ingeo Biopolymer 3521D [density 1.24 g/cm^3^, flow index 210 °C/2.16 kg for 14 g/10 min, and processing (melt) temperature 199 °C]. Mountain’s yam tubers were purchased in the region of Atequiza (Ixtlahuacán de los Membrillos), in Jalisco state, located in the western area of Mexico. Ethanol (EtOH, Golden Bell, Cd. Mexico, Mexico), sodium hydroxide (NaOH, 99%, Golden Bell, Cd. Mexico, Mexico), acetic acid (CH_3_COOH, 99%, Golden Bell, Cd. Mexico, Mexico), potassium iodide (KI, 99%, Sigma Aldrich, St. Louis, MO, USA), iodine (I_2_, 99.8%, Sigma Aldrich, St. Louis, MO, USA), and distilled water from Selectrum (Guadalajara, Mexico) were used.

### 2.2. Methods

#### 2.2.1. Starch Extraction

**Figure 1:**
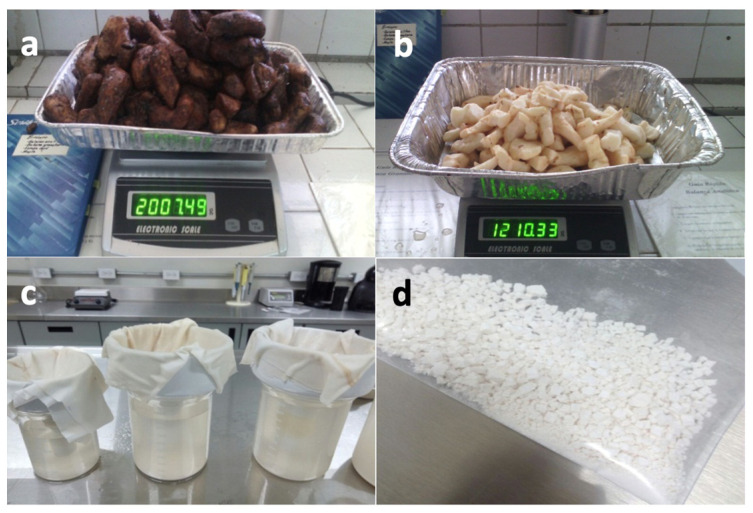
Extraction of *Dioscorea remotiflora* starch: (**a**) mountain’s yam tubers; (**b**) peeled and washed tubers; (**c**) filtration on cheesecloth of organic residues; (**d**) *Dioscorea remotiflora* starch precipitate.

Starch from *Dioscorea remotiflora* tubers was extracted according to the method proposed by Sukhija et al. [50], with some modifications (Figure 1). Tubers were washed, peeled manually with a knife, and chopped; then, the pieces were ground in a laboratory blender to coarse-grind size. The organic residues were removed by filtration on cheesecloth and the filtrate was allowed to settle to obtain the starch. The precipitate was separated and washed several times with distilled water until it was transparent. The aqueous starch dispersion was centrifuged (Labogene Multi-purpose Model 1580R; Labogene, Republic of Korea) at 2500 rpm (769 rcf) for 10 min to obtain the starch. Subsequently, the starch was dried at 40 °C for 24 h in an oven (FRELAB Model ITAM-45, FRELAB, Guadalajara, Mexico) and sieved to a particle size of 100 µm.

#### 2.2.2. Amylose Content and Yield

Starch was placed (0.1 g) in 100 mL volumetric flasks, and a mixture of 1 mL of EtOH with 9 mL of 1 N NaOH was added. The sample was heated in a boiling water bath for 15 min and then cooled to room temperature to adjust the volume to 100 mL with distilled water. Aliquots of 2.5 mL were transferred to volumetric flasks of 50 mL with 25 mL of distilled water, 0.5 mL of 1 M CH_3_COOH, and 1 mL of iodine solution (0.2% I_2_ + 2% KI). The absorbance was read at a wavelength of 620 nm, and distilled water was used as a blank. The amylose content was determined based on a calibration curve at different amylose concentrations [51]. The starch yield (*SY*), expressed as (%), was calculated as follows [50]:

(1) $$SY=\frac{m_{starch}}{m_{tuber}}\times 100$$

where *m_starch_* and *m_tuber_* represent the weight of the isolated starch and fresh tuber, respectively.

#### 2.2.3. Biocomposite Preparation

To prepare the PLA/starch biocomposites, a batch mixing process was selected instead of a continuous mixing process to improve the control of the resulting blend and to improve the control of the mixing time. Blends of PLA and starch were prepared in a Haake Rheocord Fision Model 9000 batch mixer with a 60 cm^3^ chamber capacity and roller rotors. First, 50 g of PLA was introduced into the equipment chamber at 30 rpm and 160 °C for 3 min; consecutively, the biocomposites were prepared by adding different percentages of starch (7.5, 15.0, 22.5, and 30.0 wt.% (dry basis)). After 6 min, the biocomposites were immediately collected in a container and then dried at 25 °C. On the other hand, to preserve the shape and structure of the starch granules in the PLA matrix, the biocomposites were processed using compression molding to avoid applying further high shear stresses to the starch granules, such as injection molding, which introduces high pressures into the mold. A Carver thermocompression molder (Carver, Inc., Wabash, IN, USA) was used to make 3 mm thick, 13.5 × 13.5 cm plates.

**Figure A1:**
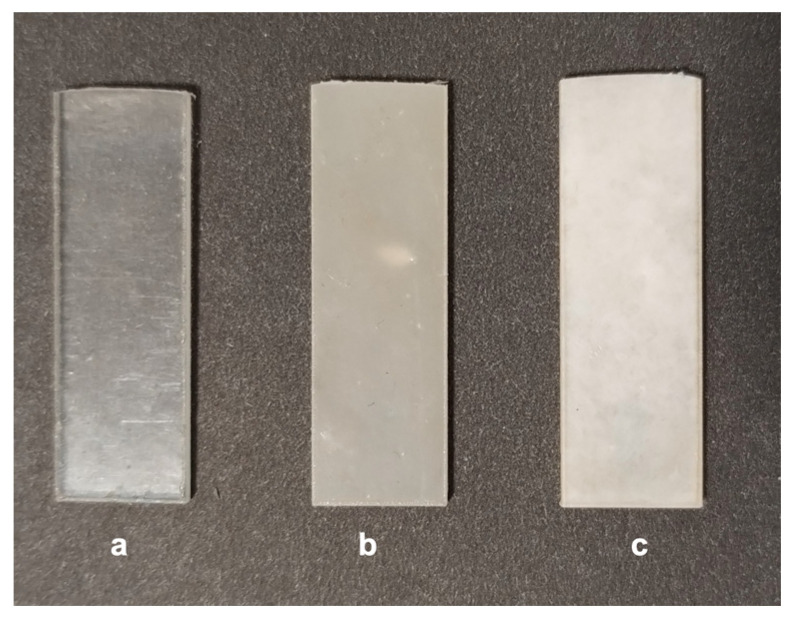
A photograph of PLA/starch biocomposites at different starch contents (wt.%): (**a**) 0; (**b**) 15; (**c**) 30.

The procedure was as follows: (1) the samples of biocomposites were annealed in the molder for 3 min and 100 bar pressure at 160 °C; (2) the pressure was released and increased to 200 bar; (3) the pressure was released for a second time and increased to 200 bar. This procedure was repeated three times, maintaining a pressure of 200 bar at 160 °C for 3 min. Finally, the biocomposites formed in the Carver thermocompression were maintained at 200 bar until they cooled at 25 °C, and, consecutively, the samples were stored for three days to eliminate stresses in the materials. A photograph of the biocomposites is shown in Appendix A (Figure A1).

#### 2.2.4. Scanning Electron Microscopy

Scanning electron microscopy (SEM) analysis was utilized to obtain micrographs of the starch granules, neat PLA, and starch biocomposites. The samples were frozen in liquid nitrogen and then fractured and coated with gold before imaging (SPi, West Chester, PA, USA) [52]. The morphology of the native starch was obtained in a TESCAN MIRA 3LMU scanning electron microscope (SEM; TESCAN, Brünn, Czech Republic) with a voltage of 15 kV. The morphology of the neat PLA and PLA/starch biocomposites was examined using a Hitachi TM-1000 field emission scanning electron microscope (Hitachi, Tokyo, Japan). The micrographs were analyzed by Image-Pro version 4.5 software (Media Cybernetics, Rockville, MD, USA). The values reported represent the average and standard deviation (*SD*) of at least 50 granules.

The starch granules were measured from the SEM micrographs to obtain the histogram and the particle size distribution (*PSD*) to analyze their dimensions more accurately. Since the starch granules have an ellipsoid-like shape, it is possible to measure the half-axes of *a* (height) and *b* (width) for each granule to estimate an average particle size (*D_i_*), defined as *D_i_* = (*a*^2^ + *b*^2^)/2. *PSD*, defined as (*D_w_*/*D_n_*), where *D_w_* and *D_n_* are the weight-average particle diameters and the number-average particle diameters, respectively, is based on the assumption that the particles are spherical, which were calculated as follows [53]:

(2) $$D_{w}=\frac{\sum_{i}n_{i}D_{i}^{4}}{\sum_{i}n_{i}D_{i}^{3}}$$

(3) $$D_{n}=\frac{\sum_{i}n_{i}D_{i}}{\sum_{i}n_{i}}=\frac{\sum n_{i}D_{i}}{n}$$

where *n_i_* is the number of particles. Moreover, the z-average particle diameter (*D_z_*_0_) was estimated according to the following Equation [54]:

(4) $$D_{z0}=\frac{\sum_{i}n_{i}D_{i}^{7}}{\sum_{i}n_{i}D_{i}^{6}}$$

#### 2.2.5. FTIR-Attenuated Total Reflectance

The Fourier Transform Infrared Spectroscopy (FTIR) analysis of the modified surface of the biocomposites was performed using a Thermo Scientific iS5 Nicolet (Thermo Fisher Scientific, Madison, WI, USA) with attenuated total reflectance (ATR). The spectra were obtained at a 4 cm^−1^ resolution, with 64 scans in the standard wavenumber range from 400 cm^−1^ to 4000 cm^−1^. The samples analyzed were oven-dried (FRELAB Model ITAM-45, FRELAB, Guadalajara, Mexico) at 50 °C for 24 h before testing [10].

#### 2.2.6. Thermal Analysis

The Differential Scanning Calorimeter (DSC) measurements were carried out using a TA Discovery Model Q100 (TA Instruments, New Castle, DE, USA). All samples were dried for 24 h in an oven at 60 °C before the analysis. The PLA and biocomposites were heated from 0 to 200 °C and maintained at 200 °C for 1 min to remove internal moisture and small volatile molecules for the first temperature scan. For the second scan, the samples were cooled to 0 °C, held for 1 min, and subsequently heated to 200 °C and held for 1 min before being cooled to 0 °C; both heating and cooling rates were at 5 °C/min during the scans [55]. For starch, a similar procedure was performed with a single temperature scan.

#### 2.2.7. X-ray Diffraction

The X-ray diffraction pattern (XDR) analysis was determined in a theta–theta diffractometer system Stadip (STOE & Cie GmbH, Darmstadt, Germany) equipped with a copper source, operating at 30 kV and 15 mA (K*α* = 1.5406 Å) at a scattering angle (2θ) range of 5–80°. The dried samples were ground to a fine powder and then fixed on a glass slide with Vaseline, which would not interfere with the measurement of the sample. The crystallinity of the biocomposites was analyzed as the ratio of the crystalline starch content in the PLA matrix to the total amount of the composite material. The crystallinity index (*IC*) was calculated with the following Equation [56]:

(5) $$IC=\frac{I_{Cr}-I_{Am}}{I_{Cr}}\times 100$$

where *I_Cr_* is the intensity of the maximum diffraction peak, measured as the height of the crystalline diffraction peak at 2θ = 16.2°, where this peak represents both the crystalline and amorphous materials, and *I_Am_* is the height of the smaller diffraction peak measured at 2θ = 18.1° related to the crystal structure of the PLA.

#### 2.2.8. Water Absorption Kinetics

Samples of 35 mm × 12 mm × 1.4 mm (*l* × *w* × *d*) were placed in a Terlab oven (TE-H35D, Terlab, El Arenal, México) at 60 °C for 24 h to eliminate the water absorbed during the preparation process. Subsequently, the samples were weighed and placed in containers with distilled water at 25 °C [57]. Samples were weighted daily for water absorption intake until they reached a constant weight. The data were registered and plotted [(moisture (%) vs. time (days)] to determine the kinetic behavior of the water absorption. Water diffusion through the lineal section of the plots was calculated using the following Equation [57]:

(6) $$\frac{M_{t}}{M_{\infty }}=k\times t^{n}$$

where *M_t_* is the absorbed moisture (%) at time *t* (s), $M_{\infty }$ is the maximum moisture concentration at infinite time (saturation), and *n* and *k* are the kinetic constants.

The coefficients of the equation (*n* and *k*) can be determined from the slope (*n*) and the intercept (*k*) by plotting the log $\left(M_{t}/M_{\infty }\right)$ vs. log *t.* From these two coefficients, the exponent *n* is of main interest to determine the type of diffusion that occurs. According to the value of *n*, the diffusion can be classified using Fick’s Law as follows: Case I: *n* = 0.5; Case II: *n* = 1; Super Case II: *n* > 1; non-Fickian or abnormal diffusion: 0.5 < *n* <1 [58]. The solution of the diffusion Equation, following Fick’s Law for isotropic media with a constant coefficient and only on one axis (x), has been reported elsewhere [58,59] as follows:

(7) $$M_{t}=M_{\infty }\left[1-\frac{8}{\pi^{2}}\sum_{n=0}^{\infty }\frac{1}{(2n+1{)}^{2}}exp\left[\frac{-D_{x}\times t}{h^{2}}\pi^{2}(2n+1{)}^{2}\right]\right]$$

where *h* is the thickness of the plate (under dry conditions) and *D_x_* is the diffusion coefficient on the *x*-axis at time *t*. The measurements were performed at least three times, reporting average values.

#### 2.2.9. Mechanical Properties

The biocomposite samples were cut with a laser machine (Guian GN-640MS Laser Cutter; Guian, Jinan, China) at a rate of 5 mm/min with a 100% cutting intensity to evaluate the samples’ mechanical properties. The tensile tests were performed in an Instron Model 3345 Universal Testing Machine (Instron, Norwood, MA, USA), utilizing a 1 kN electronic load cell and mechanical clamp grips. The measurements were performed following the standard procedure (ASTM D638 (2001)) [60] at a crosshead rate of 1 mm/min and a distance between mechanical clamp grips of 25.4 mm. The flexural test samples were carried out following the standard procedure of the ASTM D790 (2001) [61] for plastic materials with and without reinforcement, using a three-point contact system, in the Universal Testing Machine at 1 kN at a rate of 1 mm/min.

#### 2.2.10. Dynamic Mechanical Analysis

Dynamic mechanical characterization was performed in a Perkin Elmer Model DMA7 dynamic mechanical analyzer (Perkin Elmer, Waltham, MA, USA). Rectangular samples of 15 mm × 2 mm × 3 mm (*l* × *w* × *d*) were cut with a laser machine (Guian GN-640MS, Laser Cutter, Guian, Jinan, China) at a 5 mm/min speed and with a 100% cutting intensity. Temperature ramps were performed ranging from 30 to 120 °C at a constant frequency, strain, and ramp heating of 5 rad/s, 0.025%, and 5 °C/min, respectively.

#### 2.2.11. Color

The color of the blends was measured with a CR-410 Colorimeter (Konica Minolta, Ramsey, NJ, USA). Six measurements were taken at random on the surface of the samples, and the readings were recorded with the CIELAB color space to obtain the luminosity (L*) and the color space parameters a* and b*, which are related to rectangular coordinates in the color plane. Also, the total color difference (Δ*E*), which is the distance between two points within the color space parameters with respect to the control, was determined with the following Equation [62]:

(8) $$\Delta E=\sqrt{{\left({∆L}^{*}\right)}^{2}+{\left({∆a}^{*}\right)}^{2}+{\left({∆b}^{*}\right)}^{2}}$$

where ΔL*, Δa*, and Δb* represent the color space parameter differences between the sample and the control. The color measurements were replicated at least three times, and an average value was calculated.

#### 2.2.12. Statistical Analysis

Experimental data were statistically analyzed with the Statgraphics Centurion XV version 15.2.06 software (Statpoint Technologies, Warrenton, VA, USA). The comparison of the mean ± SD values between the treatments was performed with the LSD (Least Significant Difference) Fisher multiple range test at a confidence level of 95% (*p* < 0.05).

## 3. Results and Discussion

### 3.1. Starch Yield

Tubers of mountain’s yam are a rich source of starch with a yield of 21.82% *d.b.*, indicating that this type of tuber could be an alternative source of starch; other varieties of *Dioscoreas* have lower percentages, such as *Dioscorea trifida* with 20.6% or *Dioscorea pyrifolia* with 26.64% [63,64].

### 3.2. Amylose Content

The native starch contains 48.43 ± 1.4% of amylose, thus this may be classified as high amylose [based on the amylose content, starches are classified as waxy (0–2%), low (5–20%), intermediate (20–30%) and high (>30%) [65]. Tubers of species like *Dioscorea mexicana* Schidw contain 31.1% or *Dioscorea pyrifolia* of high amylose with 44.47% [63,66], but in this work, *Dioscorea remotiflora* had the superior content; on the contrary, cereals like wheat and corn are low in amylose (8.84 and 10.41%, respectively) [67], but this also depends on the cultivar.

### 3.3. Morphology

**Figure 2:**
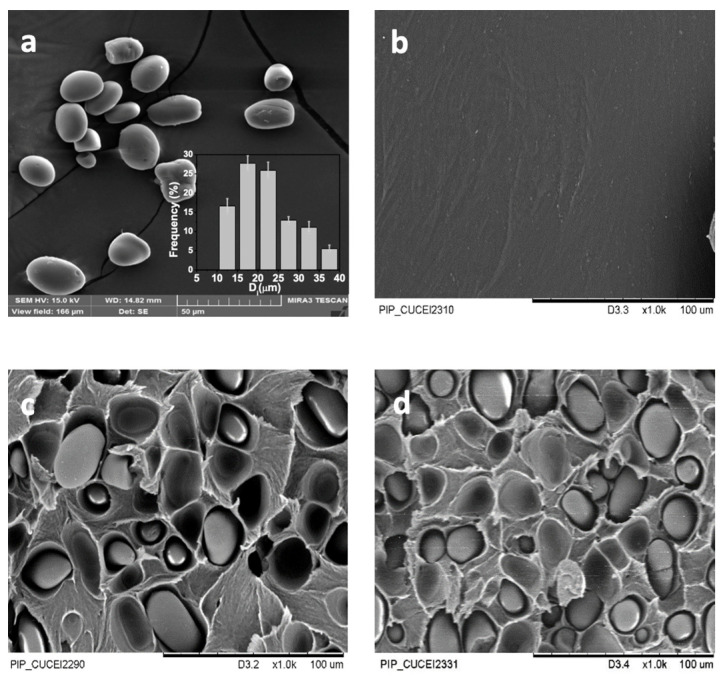
SEM micrographs of the following: (**a**) starch granules from mountain’s yam (*Dioscorea remotiflora* Kunth) and the fracture surface of (**b**) neat PLA and PLA/starch biocomposites at different starch contents of (**c**) 15 and (**d**) 30 wt.%. Inset in (**a**): SEM histogram of native starch granules from mountain’s yam (*Dioscorea remotiflora* Kunth).

Figure 2 shows the SEM micrographs of native starch granules (Figure 2a), the stress fracture of the cross-section of the neat PLA (Figure 2b), and the PLA/starch biocomposites for 15 and 30 wt.% of starch (Figure 2c,d). Figure 2a displays the starch granules from mountain’s yam (*Dioscorea remotiflora* Kunth), which are oval-like, with a smoother and flatter surface. A similar morphology has been reported for the starch granules of potatoes, peas, or green beans, as reported elsewhere [47]. The inset in Figure 2a depicts the histogram of the native starch granules. It is observed that the *PSD* is narrower (1.27 ± 0.05). The particle size analysis revealed that the *D_w_*, *D_n_*, and *D_z_*_0_ values for the starch granules are 27.15 ± 3.39, 21.25 ± 3.64, and 30.59 ± 3.44 μm, respectively, which are larger than other varieties of *Dioscorea* tubers [68,69]. According to a classification reported elsewhere, hard-cooking yam varieties have large starch granules (ca. 35 μm) [70]. Thus, larger starch granules have greater resistance to shear stresses due to dipole–dipole attractions between hydrogen-bonding forces and the formation of a double helix between the amylopectin and amylose chains [71]. In addition, they have a higher amylose content, as shown in the last section, which is an important property of starch that confers improved mechanical properties to the biocomposites. Figure 2b presents the neat PLA, where a smoother surface is shown, whereas Figure 2c,d depicts the presence of intact starch granules that are well embedded into and distributed in the matrix without apparent damage or agglomeration. Likewise, empty cavities are observed in the matrix because the starch granules were snatched by the fracture. This behavior suggests that the PLA did not exhibit complete compatibility with the starch granules in some parts. This is because the starch is highly hydrophilic and the PLA is hydrophobic, which hinders compatibility [48]. Moreover, note that an increase in starch granules to 30 wt.% does not affect the size and structure of the granules.

### 3.4. Infrared Spectra

**Figure 3:**
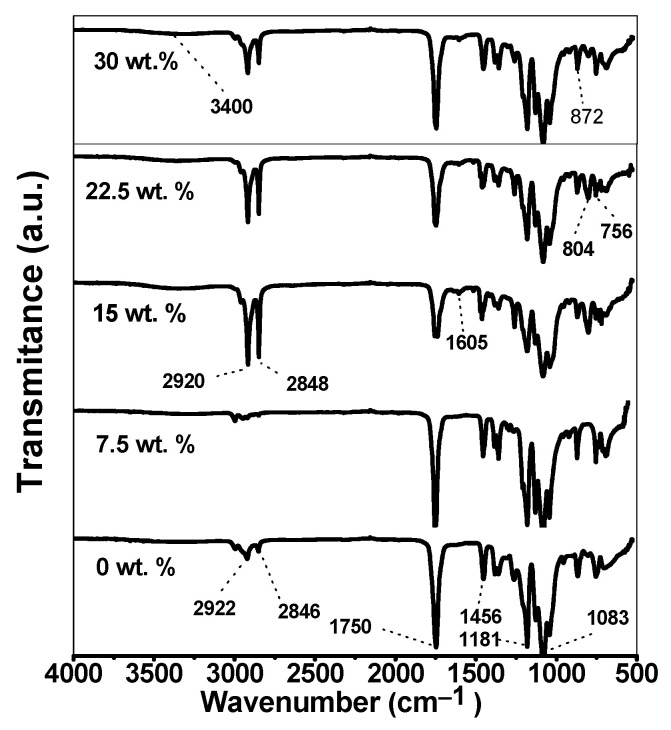
Infrared spectra of the neat PLA and PLA/starch biocomposites at different starch contents.

**Figure A2:**
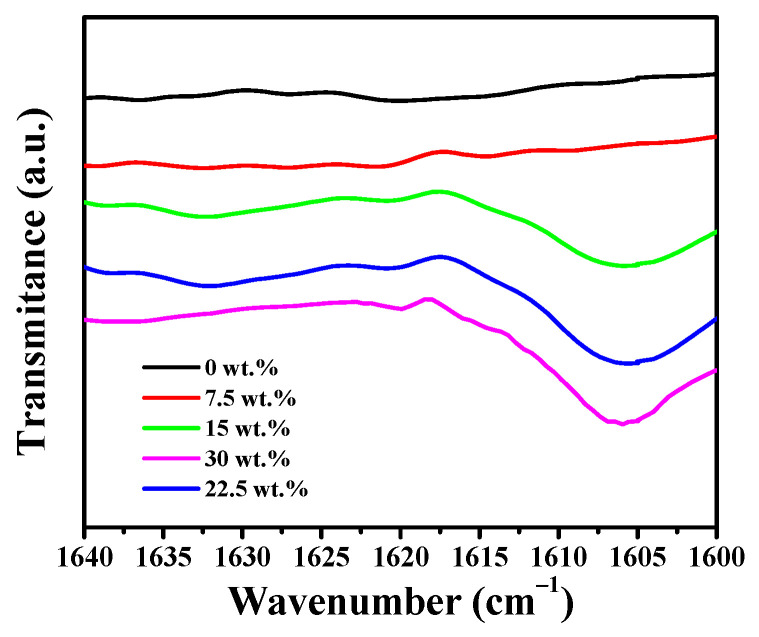
A zoom-in of the infrared spectra of PLA/starch biocomposites at different starch contents.

Figure 3 presents the infrared spectra of the PLA and the biocomposites. The infrared spectra displayed vibrations within a range of 4000–500 cm^−1^. All samples exhibited a peak at 1750 cm^−1^, which is related to the stretching vibration of the –C=O group of the PLA. Stretching vibrations corresponding to the CH2– and CH3– groups of the PLA were identified near the signals of 2920 and 2848 cm^−1^ [72]. Starch in the biocomposites was identified at starch concentrations higher than 15 wt.%. The slight band observed at 3400 cm^−1^ is related to the OH– groups due to the presence of starch, suggesting that the water present in the starch is probably linked by hydrogen bonding to the carbonyl ester group [73]. As this band was not observed for the PLA sample, nor for the 7.5 wt.% composites, this indicates that both samples were dry when analyzed. Moreover, at 1605 cm^−1^, a tiny peak related to the C–O bending group of the starch associated with the OH– group was observed for the blends at starch concentrations higher than 15 wt.% [74], which is also related to the water present in the starch [31]. These behaviors suggest that the two components are well mixed according to Yang et al. [50]. A zoom-in of this peak is reported in Appendix A (Figure A2). Other vibrations detected within the range of 1396–1417 cm^−1^ are related to the CH– bonds of the starch. The vibration band between 865 and 1083 cm^−1^ corresponds to a C–O functional group of the starch. Furthermore, the vibration of the C–O–C ring on the starch produces an absorbance peak at 756, 804, and 872 cm^−1^, as reported for three different varieties of starch [74].

### 3.5. Thermal Properties

**Figure 4:**
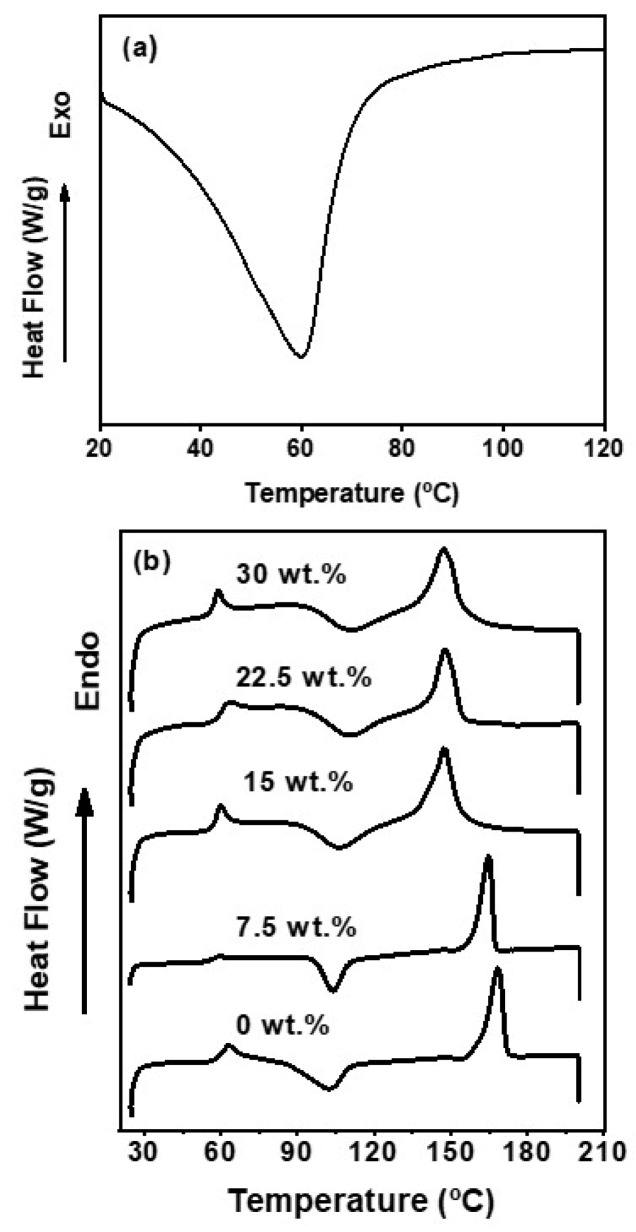
Thermograms of: (**a**) starch granules from mountain’s yam (*Dioscorea remotiflora* Kunth); (**b**) PLA/starch biocomposites at different starch contents.

Figure 4 shows the thermograms of the mountain’s yam starch and the biocomposites. A solitary peak is observed, indicating the endothermic transition phase of the biopolymer at 59.96 °C (Figure 4a), which corresponds to its glass transition temperature (T_g_). This observation is consistent with the typical T_g_ values of other starches, such as rice starch, which fall within the range of 35–70 °C [75]. Additionally, the broader endothermic peak with a singular onset temperature suggests increased homogeneity in the starch [76]. The absence of the exothermic crystallization peak (T_c_) and the endothermic melting peak (T_m_) is attributed to the slow crystallization process of amylose and amylopectin, which are large molecules constituting starch [77].

The thermal behavior of the PLA/mountain’s yam starch biocomposites is shown in the thermogram (Figure 4b). The data of the T_c_, T_m_, the crystallization enthalpy (∆H_c_), and the melting enthalpy (∆H_m_) obtained from the DSC studies are summarized in Table 1. In this table, the IC and T_g_ were measured from the XDR and DMA tests, respectively, because of the accuracy of the methods. In Table 1, an increase in the T_g_ can be observed when the mountain’s yam starch is incorporated into the PLA matrix. These observations indicate that a higher T_g_ consequently promotes a change from soft and flexible to hard properties, reducing the mobility of the PLA chains. The effect of the addition of starch granules on the crystallinity and T_g_ will be further discussed in more detail in Section 3.6 and Section 3.9, respectively. Regarding the ∆H_m_ and ∆H_c_, these decreased with the addition of the mountain’s yam starch because they hinder the PLA crystallization [47]. Similar results have been reported elsewhere [78]. Furthermore, variations in the starch type, origin, and composition significantly impact the structural ordering and crystalline formation, along with the concentration of the starch within the matrix [79].

### 3.6. X-ray Diffraction

**Figure 5:**
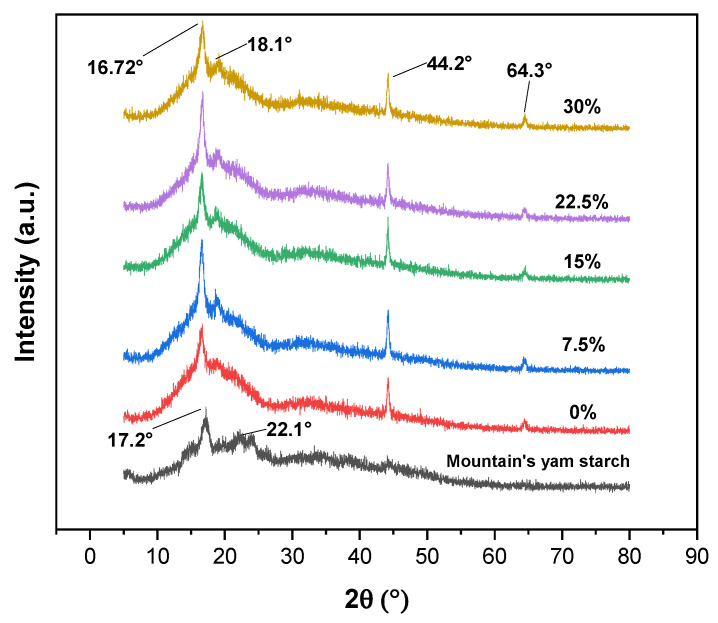
X-ray diffraction patterns of raw starch flour from mountain’s yam (*Dioscorea remotiflora* Kunth) and PLA/starch biocomposites at different starch contents.

Figure 5 shows the XDR of the native starch, neat PLA, and biocomposites. The native starch diffraction pattern displayed a typical A-type pattern at 2θ, with the first peak around 17.2°, a second peak near 19°, and the third main reflection around 22.1°. Native starch also exhibited a low-intensity peak at 20°, indicating the presence of V-type amylose–lipid complexes associated with the semi-crystalline nature of the biopolymer [80]. This XDR pattern is similar to native starches reported elsewhere [81]. The CI value of the native mountain’s yam starch was 8.82%, which is lower compared to other *Dioscoreas* yam varieties, i.e., *Dioscorea Opposita* Thunb., which contains a crystallinity percentage calculated by the XDR of 23.70 [68], and the *Dioscorea hispida* tuber with a CI value of 27.5% [69]. This result indicated that the CI value of mountain’s yam starch is lower due to the high amylose content of the starch, since, generally, the higher the amylose content in the starch, the lower its crystallinity [68]. The IC values of the raw starch flour and the biocomposites are reported in Table 1. On the other hand, the diffraction patterns of the biocomposites showed peaks at 16.2° and 18.1°, which correspond to the crystal planes of (200/110) and (203), respectively [82]. Meanwhile, the peaks at 44.2° and 64.3° correspond to the crystal planes of (200) and (220), respectively. These peaks are indicative of certain molecular arrangements of crystalline phases within the PLA [83], whereas the CI values of the biocomposites increased from 70.02 to 75.53%. These results were consistent with those reported elsewhere [84]. The starch granules were exposed to high temperatures and high shear forces during the biocomposite blending process, strongly affecting their crystalline structure and resulting in the disappearance of the diffraction peaks, as shown in Figure 5. Therefore, this suggests that the increase in the starch content increased the relative crystallinity of the biocomposites.

### 3.7. Water Absorption and Kinetics

**Figure 6:**
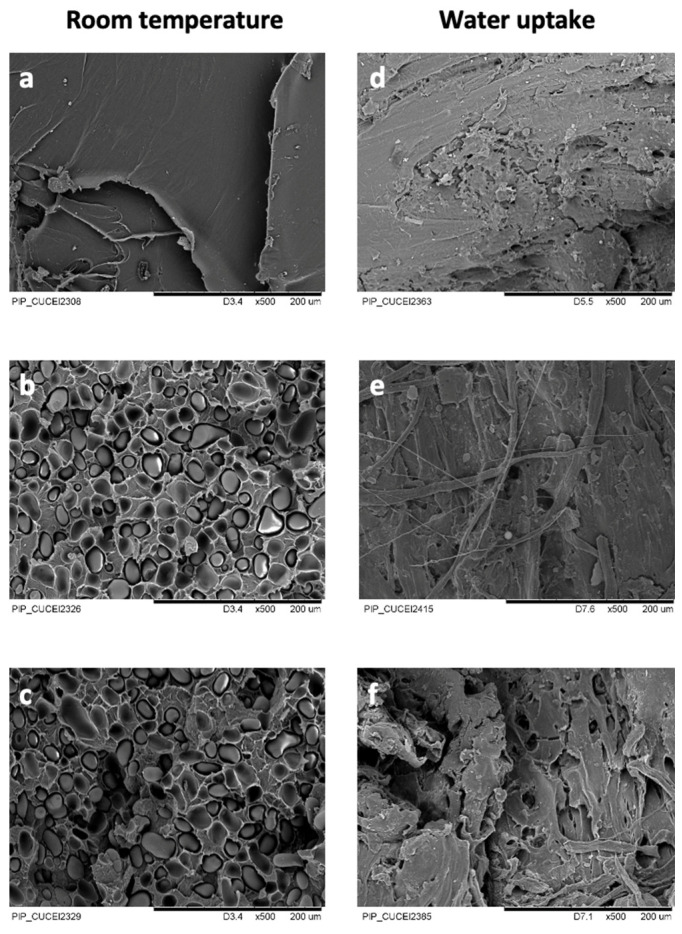
SEM micrographs of the fracture surface of PLA/starch biocomposites at different starch contents (wt.%): (**a**) 0, (**b**) 15, and (**c**) 30; wet samples (wt.%): (**d**) 0, (**e**) 15, and (**f**) 30.

Figure 6 presents the SEM images of the PLA and PLA/starch biocomposites with and without water uptake. Starch granules and hollows inside the matrix can be observed as a direct sign of adhesion (Figure 6a–c) [85]. After water uptake and the subsequent drying of the samples, the material exhibited hollows between the starch granules and PLA (Figure 6d–f). This phenomenon is related to the complete hydration of the starch granules that are withdrawn from the matrix surface, which is not sufficiently strong to retain the starch granule [86]. Moreover, solubilization of the starch in the water might have occurred, leaving those empty spaces. On the other hand, the PLA matrix presents deterioration on the surface, suggesting its hydrolytic degradation. This phenomenon induces the development of a heterogeneous surface, one that is rough with hollows, and with the presence of threads on the matrix.

**Figure 7:**
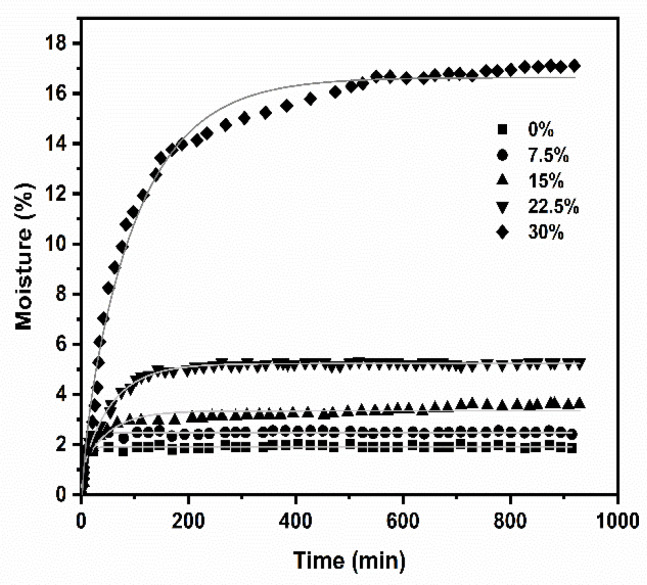
Water uptake profiles of composites of PLA/starch biocomposites at different starch contents. The solid lines are the best fit with Equation (6).

Figure 7 depicts the PLA absorption curves and PLA/starch biocomposites. As the starch content increases, the moisture content also increases due to the hydrophilic nature of the starch, which can absorb large amounts of water. The greatest effect is observed at 30 wt.% of starch, where the highest moisture is achieved, with a steeper slope reached within the first minutes. It is also observed that, at low percentages of starch, the absorption curve exhibits two zones; in the first region, there is a gradual increase in moisture, while the second region corresponds to a plateau developed for nearly the entire period. This latter behavior indicates that the hydrophilic sites of the starch reached saturation faster than samples containing higher percentages of starch, since more –OH groups are available. Therefore, the water absorption in the PLA biocomposites with starch increases due to several factors: (1) the porous structure created by the starch in the PLA matrix, which offers more binding sites for water, (2) the starch is hydrophilic and its increase in the PLA matrix increases the water–material interaction, and (3) the distribution of starch particles increases the contact surface between water and the PLA matrix, and the permeability of the material is also affected. The solid lines in Figure 7 represent the best fit of Equation (6). Table 2 presents the parameters of Fick’s Law (Equation (6)) and the moisture diffusion coefficient (*D_x_*) (calculated from Equation (7)) of the PLA and PLA/starch biocomposites. Equation (6) exhibited a good fit with the experimental data, with correlation coefficients higher than 0.9 (not shown). Regarding *n*, the values were close to 0.5, indicating that the diffusion follows the Fick Equation (Equation (6)). The PLA and the PLA/starch biocomposite with 7.5 wt.% starch yielded similar *n* values of 0.363 and 0.392, respectively; this means that low percentages of starch give rise to little effect on the swelling capacity. Otherwise, the PLA/starch biocomposites with 15, 22.5, and 30 wt.% of starch rendered values of 0.42, 0.56, and 0.849, respectively, indicating a non-Fickian diffusion process; thus, the addition of starch influences the diffusion phenomena through the biocomposites. However, understanding the starch swelling process not only involves elucidating the types of water diffusion mechanisms, but more studies are needed to understand the physicochemical reactions between the amylose and amylopectin and how they impact the structure of the starch granules and their swelling kinetics.

### 3.8. Mechanical Properties

Table 3 presents a summary of the mechanical properties of the PLA/starch blends. Statistically, the addition of starch was significant (*p* < 0.05) for all mechanical parameters. The incorporation of starch affects both the flexural (σ*_flex_*) and tensile (σ) strength as well as their respective moduli. σ*_flex_* undergoes a notable decline as the starch content increases to 30 wt.% (45.02 MPa), in contrast to the pure polymer (72.89 MPa), while the maximum σ decreases significantly to 62.73 MPa compared to 79.11 MPa for the neat PLA. This decrease indicates that the addition of the starch does indeed reduce the adhesion between the blended materials; that is, it contributes to stiffness without reinforcing the PLA, meaning that the starch modifies the properties of the PLA [87,88,89]. Additionally, incorporating starch into the composite enhances the flexural modulus (E_flex_), reaching a peak value of 1868.73 MPa at a critical starch concentration of 22.5 wt.%, implying an effective stiffness transfer to the PLA matrix. However, once the critical concentration of starch is exceeded, the E_flex_ value decreases, probably due to the poor adhesion and compatibility between the starch and PLA. For the tensile modulus (E), its behavior was similar to that of the maximum resistance to tension, since the values also decreased with the increasing starch content; thus, the higher the percentage of starch, the lower the value of E. The poor adhesion between the starch and the polymeric matrix is attributed to the high hydrophilicity of the starch, which reduced the σ exhibited by the biocomposites. Therefore, the starch in the blend behaves as a filler because of the composition of starch, the ratio of the starch particles, and their irregular oval shape. This situation promotes poor interaction between the starch and the matrix. However, the values of σ are higher than those of the PLA biocomposites prepared with starch granules from different botanical sources, but with similar sizes and shapes to those extracted in this work [47]; meanwhile, the E values are lower than those prepared with potato starch (1470 MPa). Khalid et al. [47] reported tensile strength values of 40.34 ± 3.77, 31.44 ± 2.80, and 28.85 ± 3.38 MPa for PLA composites with 30 wt.% of starch granules from potato (granule size of 24.93 ± 8.96 μm), sweet potato (granule size of 15.54 ± 11.09 μm), and pea (granule size of 18.22 ± 6.22 μm), respectively. Note that the starch granules of the *Dioscorea remotiflora* tuber are larger than those of potato and yet have higher σ values. In general, small particle sizes increase the tensile strength due to the reduction in the stress concentration sites in the PLA matrix [90]. However, these results may be due to multiple factors, such as the amylose content in the starch [5,47], the interfacial forces between the particle and the matrix [91], the particle size [92] and shape [47], among others. As demonstrated here, the amylose content in the mountain’s yam starch is higher compared to the amylose content of potato (20%) [22], suggesting that the improved E_flex_ values are due to the high amylose content. The amylose plays a fundamental role in the mechanical properties of the composites, i.e., the PLA/starch blends prepared with high-amylose starch have better elongation at break and tensile strength compared to those materials prepared with low-amylose starch [27]. Similarly, extruded thermoplastic films of starch high in amylose presented better mechanical properties and high-impact strength than films with starch low in amylose [28]. Moreover, it has been reported elsewhere that the deformation of composites was improved using high-amylose starches [47]. Nevertheless, further research is required to understand the multiple factors that exert an influence on the mechanical properties of the composites together with the *Dioscorea remotiflora* starch.

### 3.9. Dynamic Mechanical Properties

**Figure 8:**
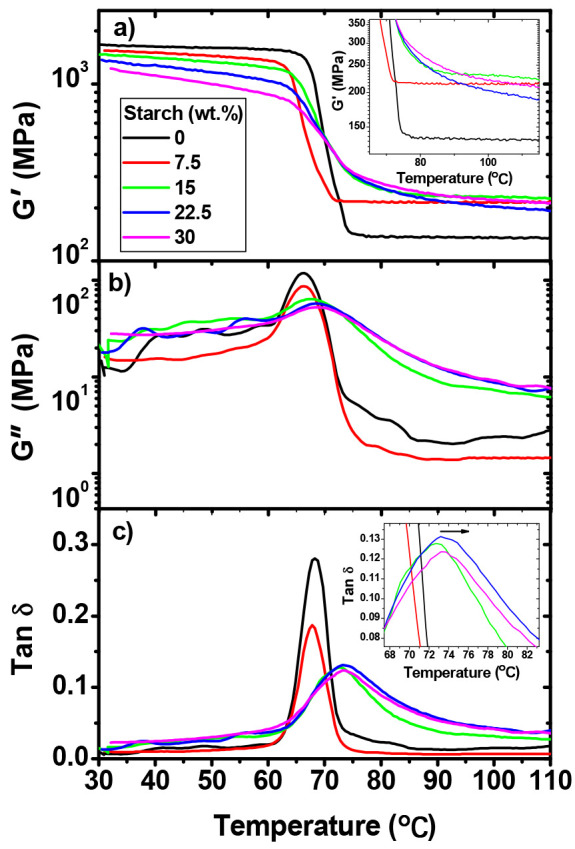
Temperature dependence of the (**a**) storage modulus (G′), (**b**) loss modulus (G″), and (**c**) tan δ for PLA/starch biocomposites at different starch contents. Insets: zoom-in of the storage modulus versus temperature (**a**) and tan δ versus temperature (**c**) for PLA/starch biocomposites at different starch contents.

The storage modulus (G′), the loss modulus (G″), the tan δ of the PLA, and the biocomposites of the PLA starch are shown in Figure 8. Figure 8a reveals that the G′ values of the PLA and the biocomposites decrease with increasing temperature due to the increase in the polymer chain mobility of the matrix. The drop in the modulus is abrupt near the glass transition of the PLA between 62 and 75 °C, where the T_g_ is located. Also, the glassy plateau modulus decreased by increasing the starch content in the biocomposites, indicating a lower stress-transfer efficiency due to the starch granules. This result is consistent with the tensile modulus reported previously (Table 3). As can be noted in Figure 8a (inset), the rubbery plateau modulus is larger for the PLA/starch biocomposites than for that of the neat PLA, suggesting that the biocomposites become more crystalline. The rubbery plateau modulus exhibits more limited motion related to its amorphous state due to the increased crystallinity, which causes an increase in G′ in the rubbery region.

Figure 8b depicts the variation in the loss modulus with the temperature of the PLA and the biocomposites with the different starch loads. The peak height of PLA and biocomposites at a starch content of 7.5 wt.% shows a consistent reduction as the starch content increases. Conversely, when the starch content ranges from 15 to 30 wt.% within the PLA matrix, a notable broadening of the loss modulus curve is observed. This broadening phenomenon can be attributed to variations in the physical state of the starch-containing region compared to the rest of the matrix, as the polymer layer encompassing the starch increases, resulting in an increased volume fraction of the matrix, intensifying the interfacial restrictions [93]. Consequently, these constraints exert a pronounced influence on the amorphous phase, potentially resulting in a more pronounced or broader glass transition behavior.

From Figure 8c, it is possible to identify the value of the tan δ (alpha-transition) peaks of the PLA and the biocomposites. The tan δ peak for the neat PLA was observed at approximately 68 °C, while, for the biocomposites, the tan δ peaks were measured at approximately 67 °C for those containing 7.5 wt.% of starch, and these peaks increased to 72 and to 73 °C for those containing 15 and 30 wt.% of starch, respectively. Therefore, this suggested a restrictive effect on the movement of the molecular segment. These results are indicative of the composites becoming more crystalline due to the presence of starch [93]. An increase in the T_g_ means that more thermal energy is required to induce the motion of the polymer backbone, which is chained by the crystalline phase, as it passes through the glass transition region [93,94]. Khalid et al. [47] reported similar results, where the T_g_ increased in the PLA matrix with the addition of starch granules from different botanical sources. The authors attributed this result to the reduced mobility of the polymer chains due to the starch granules.

### 3.10. Color Parameters

The CIELab color space method is one of the most widely used methods to evaluate the color of objects or materials and to correlate numerical color values consistently with human visual perception. Luminosity (L*) indicates how opaque or luminous (not related to brightness) a surface is, and their values range from 0 to 100 [0 = black; 100 = white]. Statistically, in Table 4, the addition of starch exerted a significant effect (*p* < 0.05) for this parameter. Neat PLA displayed the highest L* value of 82.97, in comparison with the blends that were within a close range of 70.22–74.18 (Table 4), which is narrow since the starch increase was quite considerable from 7.5 to 30 wt.%; therefore, this suggests that starch, independently on the amount added, losses its birefringence at high temperatures, becoming opaque and reducing the luminosity of the samples (Figure A1). Hence, a* goes from red to green (+a*: red and −a*: green) and b* goes from yellow to blue (+b*: yellow and −b*: blue), a representation that facilitates analysis in the color plane. For all biocomposites and the neat PLA, both a* and b* were in the positive region, tending toward red and blue, respectively. Neat PLA exhibited the lowest values of both color parameters, which significantly (*p* < 0.05) increased with the addition of starch. Although there was a considerable difference in the order of magnitude between a* and b*, among the biocomposites, values did not increase drastically with the increase in the percentage of starch >7.5 wt.% for a* (1.08–1.53); nonetheless, the wider variation range of b* (10.97–13.50) could be more useful to predict color changes in starch-based blends, since this parameter had larger increases than a*. On the other hand, the total color difference (Δ*E*), calculated from Equation (8), defined as the numerical comparison of a sample with the standard, also increased with the increase in starch. All Δ*E* values of the blends were above 3.0 [Δ*E* < 1.5 is not noticeable with the naked eye, from 1.5 to 3 is barely noticeable, and Δ*E* > 3.0 is visually detectable] [95], meaning that color changes are visually noticeable on sight, even at a low starch content. Thus, the changes observed in all color parameters were induced by the high temperatures applied during the mixing of both materials (starch and PLA), in that starch is a biopolymer highly susceptible to heat. Nonetheless, the opacity exhibited by the starch blends could comprise an advantage in the development of packaging for food products containing compounds, such as vitamins or antioxidants, that are sensitive to light exposure.

## 4. Conclusions

Here, it was shown that PLA biocomposites blended with high-amylose starch granules extracted from the underexplored mountain’s yam (*Dioscorea remotiflora*) tubers exhibited larger water uptake, faster swelling kinetics, a higher crystallinity, and an improved flexural modulus compared to the neat PLA, and can be easily fabricated without the need for functionalization agents or compatibilizers. By varying the starch granule content, not only does the material become more crystalline, but the water uptake exhibits the following two swelling diffusion mechanisms: a Fickian diffusion at a low starch granule concentration (<15 wt.%) and a non-Fickian or abnormal diffusion at higher concentrations (>15 wt.%). These properties are attributed to the high amylose content and the bigger size of the starch granules, which implies an effective stiffness transfer to the PLA matrix.

The investigation aimed to develop degradable bioplastics, and the starch from *Dioscorea remotiflora* tubers could be used as a promising alternative to develop low-cost bioplastics for different applications. Additionally, the opacity provided by the starch to the biocomposites after the thermoplastic process could be beneficial for packaging light-sensitive products. Further, starch granules may be functionalized with novel green chemicals extracted from plants to cross-link them and to increase their intermolecular bonds for more resistant and thermally stable granules, improving the properties of the biocomposites.

## Figures and Tables

**Table 1:** XDR, DMA, and DSC data of raw starch flour, neat PLA, and PLA/starch biocomposites.

Starch (wt.%)	^a^ IC (%)	^b^ T_g_ (°C)	* T_c_ (°C)	* ΔH_c_ (J/g)	* T_m_ (°C)	* ΔH_m_ (J/g)
Raw Starch Flour	8.82	-	-	-	-	-
0.0	65.57	68.25	102.03 ± 1.42	30.19 ± 1.82	168.37 ± 1.27	46.00 ± 1.89
7.5	70.02	67.85	104.06 ± 1.51	23.67 ± 2.1	165.00 ± 1.43	40.94 ± 1.65
15.0	71.78	72.74	106.00 ± 1.89	16.17 ± 2.63	147.22 ± 1.61	37.00 ± 1.3
22.5	73.46	73.19	110.31 ± 2.1	12.21 ± 2.94	147.89 ± 1.18	36.94 ± 1.48
30.0	75.53	73.23	109.98 ± 1.96	11.63 ± 2.25	147.23 ± 1.42	26.70 ± 1.5

* Mean value of three measurements ± standard deviation (n = 3). ^a^: The IC values were calculated from Equation (5). ^b^: The T_g_ data have been extracted from the DMA tests.

**Table 2:** Parameters of Fick’s Law and the moisture diffusion coefficient of PLA and PLA/starch biocomposites prepared with different starch contents.

Samples, (wt.%)	* *K*, (s^−1^)	* *n*	*M*_∞_, (%)	^α^ *D* × 10^−9^, (cm^2^/s)
0	−2.054	0.363	1.915	2.018
7.5	−2.494	0.392	2.411	2.001
15	−2.180	0.420	3.615	2.010
22.5	−2.156	0.569	5.212	1.932
30	−2.138	0.849	17.107	1.167

* *K* and *n* values were calculated from Equation (6). ^α^ D values were calculated from Equation (7).

**Table 3:** Flexural and tensile strength parameters of PLA and PLA/starch biocomposites.

Starch (wt.%)	Mechanical Properties			
	Flexural Strength (σ*_flex_*, MPa)	Flexural Modulus (E_flex_, MPa)	Tensile Strength (σ, MPa)	Tensile Modulus (E, MPa)
0.0	72.89 ± 1.19 ^e^	1719.32 ± 15.20 ^a^	79.11 ± 0.40 ^e^	968.35 ± 6.37 ^c,d,e^
7.5	68.50 ± 0.52 ^d^	1774.01 ± 36.35 ^b^	76.54 ± 2.21 ^d^	939.01 ± 30.20 ^c,d^
15.0	57.98 ± 1.32 ^b,c^	1792.08 ± 10.82 ^b,c^	68.04 ± 2.24 ^c^	914.73 ± 38.84 ^b,c^
22.5	55.32 ± 2.21 ^b^	1868.73 ± 44.97 ^d^	61.88 ± 1.78 ^a^	845.23 ± 14.89 ^a^
30.0	45.02 ± 2.47 ^a^	1800.96 ± 47.80 ^c,d^	62.73 ± 1.11 ^a,b^	849.45 ± 37.20 ^a,b^

Superscripts with different letters indicate a significant statistical within columns, with the LDS (Least Significant Difference) statistical test at a confidence level of 95.0%.

**Table 4:** CIELab color parameters of PLA and PLA/starch biocomposites.

Treatment	Starch (wt.%)	L*	a*	b*	*^b^*Δ*E*
1	0.0	82.97 ± 0.70 ^a^	0.63 ± 0.03 ^a^	3.58 ± 0.13 ^a^	-
2	7.5	74.18 ± 0.15 ^b^	0.68 ± 0.10 ^b^	9.66 ± 0.12 ^b^	10.69
3	15.0	72.41 ± 0.37 ^b,c^	1.08 ± 0.09 ^c^	10.97 ± 0.07 ^b,c^	12.91
4	22.5	71.98 ± 0.52 ^c,d^	1.27 ± 0.03 ^d^	12.16 ± 0.32 ^d^	13.86
5	30.0	70.22 ± 0.24 ^e^	1.53 ± 0.04 ^e^	13.50 ± 0.42 ^e^	15.39

Superscripts with different letters indicate a significant statistical difference between treatments, with the LDS (Least Significant Difference) statistical test at a confidence level of 95.0%. *^b^*Δ*E* values were calculated from Equation (8).

## Data Availability

Data are contained within the article.
